# Fatty acids and lipid mediators in inflammatory bowel disease: from mechanism to treatment

**DOI:** 10.3389/fimmu.2023.1286667

**Published:** 2023-10-05

**Authors:** Dong Yan, Shuyu Ye, Yue He, Sidan Wang, Yi Xiao, Xin Xiang, Minzi Deng, Weiwei Luo, Xuejie Chen, Xiaoyan Wang

**Affiliations:** ^1^ Department of Gastroenterology, The Third Xiangya Hospital of Central South University, Changsha, China; ^2^ Hunan Key Laboratory of Non-Resolving Inflammation and Cancer, Cancer Research Institute, Central South University, Changsha, China

**Keywords:** inflammatory bowel disease, fatty acid, lipid mediators, pathogenesis, therapeutic approaches

## Abstract

Inflammatory Bowel Disease (IBD) is a chronic, relapsing inflammatory disorder of the gastrointestinal tract. Though the pathogenesis of IBD remains unclear, diet is increasingly recognized as a pivotal factor influencing its onset and progression. Fatty acids, essential components of dietary lipids, play diverse roles in IBD, ranging from anti-inflammatory and immune-regulatory functions to gut-microbiota modulation and barrier maintenance. Short-chain fatty acids (SCFAs), products of indigestible dietary fiber fermentation by gut microbiota, have strong anti-inflammatory properties and are seen as key protective factors against IBD. Among long-chain fatty acids, saturated fatty acids, trans fatty acids, and ω-6 polyunsaturated fatty acids exhibit pro-inflammatory effects, while oleic acid and ω-3 polyunsaturated fatty acids display anti-inflammatory actions. Lipid mediators derived from polyunsaturated fatty acids serve as bioactive molecules, influencing immune cell functions and offering both pro-inflammatory and anti-inflammatory benefits. Recent research has also highlighted the potential of medium- and very long-chain fatty acids in modulating inflammation, mucosal barriers, and gut microbiota in IBD. Given these insights, dietary intervention and supplementation with short-chain fatty acids are emerging as potential therapeutic strategies for IBD. This review elucidates the impact of various fatty acids and lipid mediators on IBD and delves into potential therapeutic avenues stemming from these compounds.

## Introduction

Inflammatory Bowel Disease (IBD) is a gastrointestinal chronic inflammatory disorder characterized by mucosal barrier disruption, dysbiosis, and immune dysregulation, which mainly comprises ulcerative colitis (UC) and Crohn’s disease (CD). Although the etiology of IBD remains unclear, genetic susceptibility and multiple environmental factors play significant roles (1). As one of the modifiable risk factors, diet has gained increasing attention from researchers (2, 3), where fatty acids derive mostly from diet. Growing evidence demonstrated that “western diet” rich in high fat is closely associated with the risk and progression of IBD (3, 4), with supplementation exacerbating colitis severity in both IBD patients and dextran sulfate sodium (DSS) induced colitis mice (5, 6). This may be attributed to the effects of fatty acids on the regulation of inflammatory pathways, the mucosal barrier, and the intestinal microbiota, as fatty acids are essential components of dietary lipids (7).

Fatty acid is a diverse class of molecules consisting of hydrocarbon chains of different lengths and degrees of desaturation. Classification based on carbon chain length divides them into short-chain fatty acids (SCFAs), medium-chain fatty acids (MCFAs), long-chain fatty acids (LCFAs), and very long-chain fatty acids (VLCFAs) (8). LCFAs and VLCFAs are mainly derived from dietary intake, whereas SCFAs are formed by the conversion of indigestible dietary fibers by specific gut bacteria. In LCFAs, the anti-inflammatory effect of ω-3 fatty acids has been widely confirmed, while ω-6 is connected with impacts of pro-inflammation (9). ω-3 and ω-6 fatty acids can also produce lipid mediators that regulate inflammation resolution (10). SCFAs, which interact with the gut microbiota, are considered important factors in regulating physiological processes such as metabolism and inflammation in the human body (11, 12). Briefly, fatty acids vary in length and type, playing crucial roles in physiological regulation within the body.

**Figure 1:**
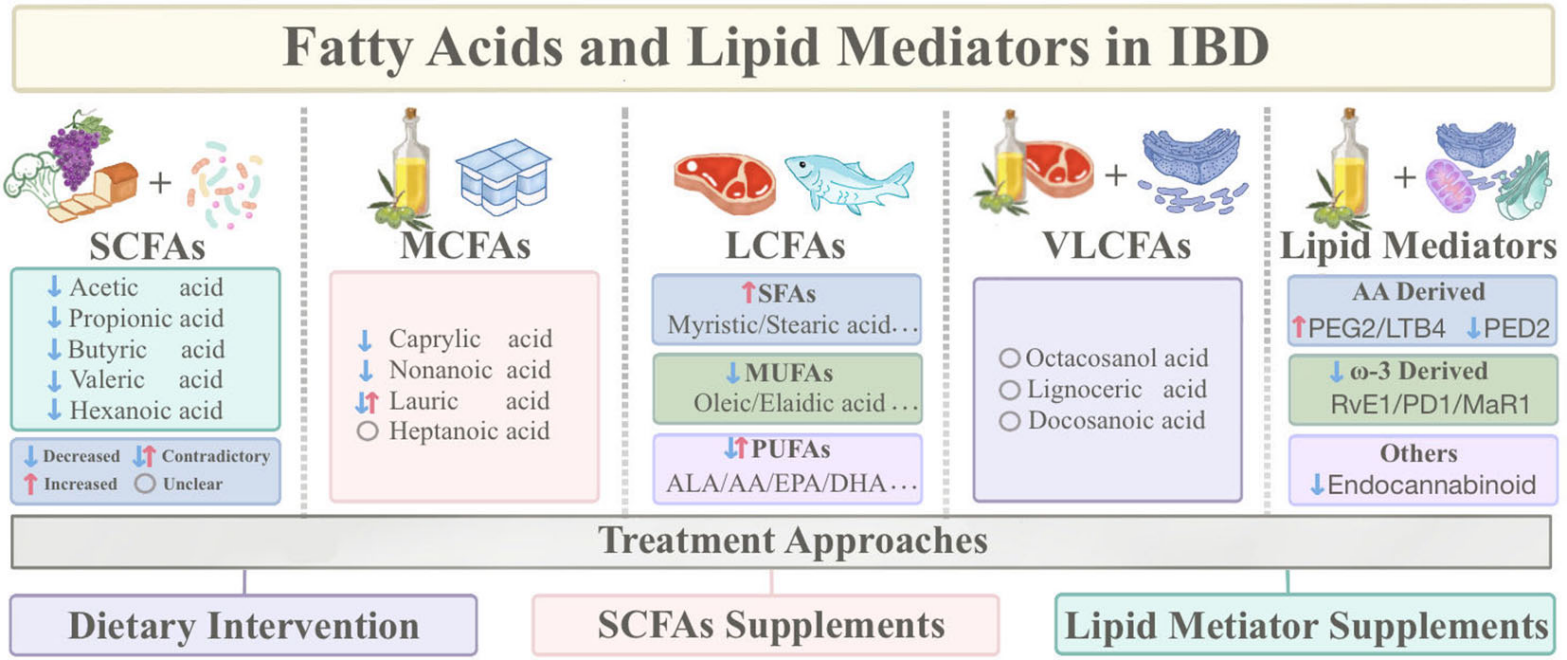
Sources of fatty acids and lipid mediators and classification based on carbon chain length, as well as therapeutic approaches based on fatty acids. (IBD, Inflammatory Bowel Disease; SCFAs, Short-chain fatty acids; MCFAs, Medium-chain fatty acids; LCFAs, Long-chain fatty acids; VLCFAs, Very long-chain fatty acids; SFAs, Saturated fatty acids; MUFAs, Monounsaturated fatty acids; PUFAs, Polyunsaturated fatty acids; DHA, Docosahexaenoic acid; EPA, Eicosapentaenoic acid; ALA, Alpha-linolenic acid; AA, Arachidonic acid).

Intriguingly, emerging research highlights the varying roles that different types of fatty acids play in the progression of IBD. SCFAs are closely connected with gut microbiota and have been reported to alleviate intestinal inflammation, maintain the mucosal barrier, and regulate immunity. On the contrary, LCFAs promote inflammation and contribute to IBD induction and aggravation. In addition, an imbalance in ω-6 and ω-3 intake is among the factors contributing to IBD onset. ω-3 polyunsaturated fatty acids are thought to be anti-inflammatory and ameliorate IBD (13), while ω-6 fatty acids are considered to contribute to IBD pathogenesis (14). Lipid mediators, derived from PUFA through at least one oxidation step, possess inflammation-regulating capabilities (10). Fatty acids and lipid mediators wield critical roles in IBD, particularly LCFAs and SCFAs, holding promise as prospective therapeutic targets for IBD. However, there is limited knowledge of what forms and amounts of fatty acids and lipid mediators should be consumed to benefit patients with IBD, and evidence-based dietary guidance is relatively scarce. Thus, this review categorizes fatty acids and lipid mediators, clarifies their roles in IBD pathogenesis, and introduces fatty acid-based therapeutic approaches (**Figure 1**).

## Short-chain fatty acids

SCFAs mainly include butyric acid, propionic acid, and acetic acid, which not only provide nourishment to the intestinal mucosal epithelium but also exhibit potent anti-inflammatory effects, and regulate immune functions (15). They also collaborate with the gut microbiota to maintain intestinal homeostasis (16). Dietary fiber serves as a substrate for SCFAs production. Gut microbiota capable of breaking down indigestible dietary fibers has the capacity to produce SCFAs (17). The inflammatory response in the gut is modulated by the intestinal microbial ecosystem, with an imbalanced microbiota being a hallmark of IBD (18). At a molecular level, SCFAs act as ligands for G-protein-coupled receptors, exerting their physiological effects (19).

Epidemiological studies have illustrated the role of SCFAs in IBD. Evidence from population-based research implies lower levels of isobutyric, butyric, propionic, and acetic acids in patients with active IBD, accompanied by diverse alterations in SCFAs patterns between CD and UC (20). The dysbiosis of the intestinal microbiota is a significant manifestation of IBD. In CD, the composition of the gut microbiota undergoes distinct changes, including the loss of SCFA-producing bacteria such as *Roseburia*, *Eubacterium*, *Subdoligranumum*, and *Ruminococcus*, alongside an increase in pro-inflammatory microbial groups (21). As a result, the diminished ability to synthesize butyrate in CD was more pronounced than in UC, potentially linked to reduced dietary fiber intake (22) and decreased butyrate-producing bacteria (23). During mucosal healing in pediatric CD, *Dialister* species and levels of butyrate salts notably increased (24). Furthermore, compared to first-time surgical patients, recurrent patients exhibited significant downregulation in levels of cyclohexanoic acid, 2-methylbutyric acid, and isobutyric acid (25), and CD patients with a history of abdominal surgery showed lower levels of butyrate-producing bacteria (26). However, Kiasat A et al. suggested that plasma SCFAs changes are unrelated to CD and UC after adjusting for gender, age, and diet (27).

**Figure 2:**
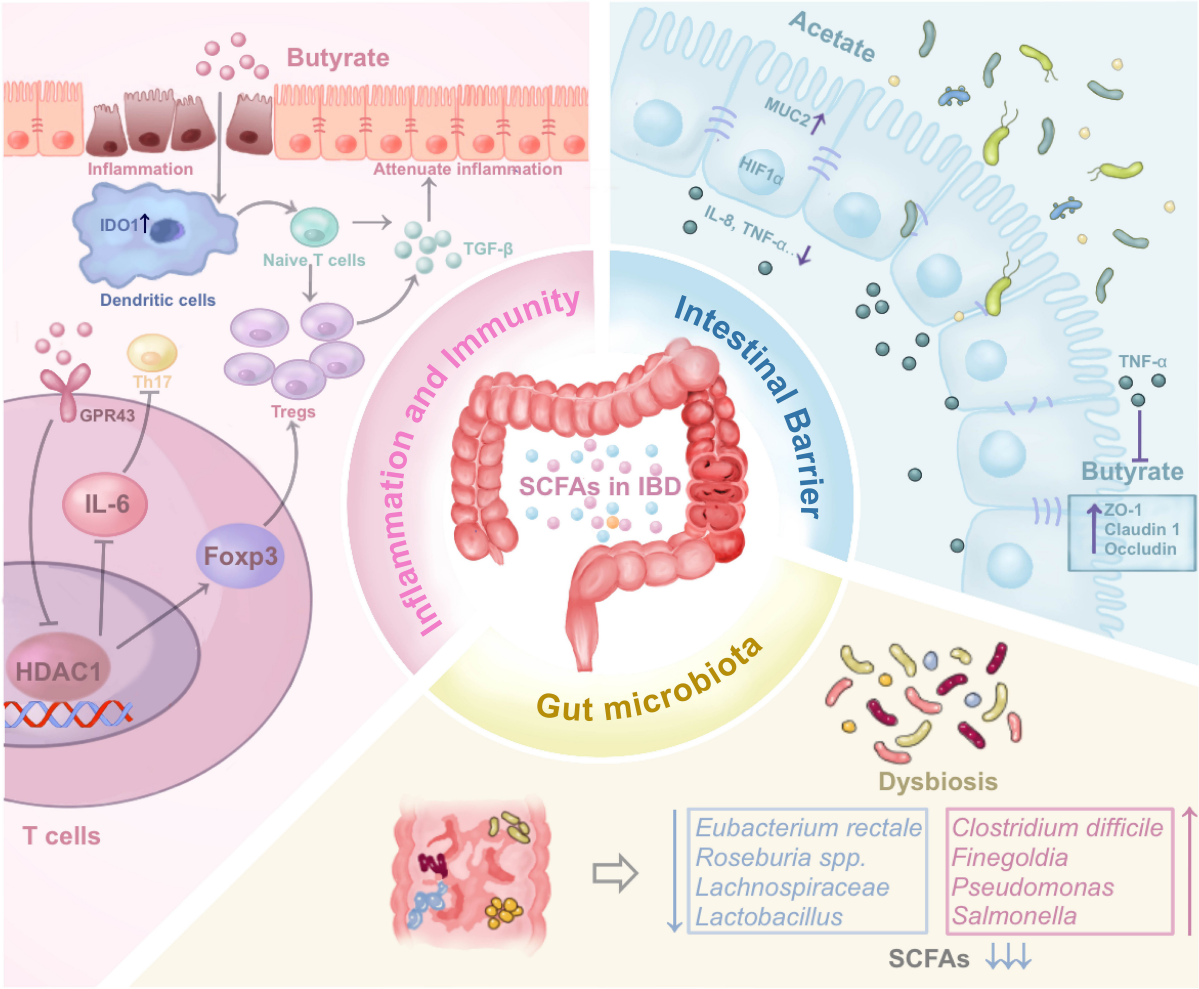
The impact of SCFAs on IBD by regulating inflammation and immune, regulating gut microbiota, and maintaining intestinal barrier through various mechanisms. The figure illustrates the three potential mechanism of SCFAs mentioned in the review. (SCFAs, Short-chain fatty acids; IBD, Inflammatory Bowel Disease; HDAC1, Histone Deacetylase 1; MUC2, Mucin 2; ZO-1, Zonula occludens-1).

The bulk of SCFAs research has predominantly centered around butyrate. Thus, we will detail the implications of SCFAs concerning IBD, encompassing facets of modulation of inflammation and immunity, regulation of intestinal microbiota, and maintenance of the intestinal barrier (**Table 1**, **Figure 2**).

**Table 1:** Mechanism of SCFAs in inflammatory bowel disease.

Fatty Acid	Animal Model	Cell	Mechanism	Author	Year
SCFAs	DSS-induced mice colitis		SCFA-GPR43 interactions modulate colitis by regulating inflammatory cytokine production in mononuclear cells	Ryuta Masui et al. (28)	2013
	SAMP1/YitFc mice		Probiotic combination (*Saccharomyces boulardii*, *Lactobacillus rhamnosus*, *Lactobacillus acidophilus*, *Bifidobacterium breve*, and amylase) ameliorates CD-like ileitis by increasing SCFA production, modulating essential adaptive immune pathways	Luca Di Martino et al. (40)	2023
Butyrate (commensal metabolite)	DSS-induced mice colitis		GPR109A activation by butyrate and niacin suppresses colonic inflammation and carcinogenesis	Nagendra Singh et al. (29)	2014
Butyric acid	DSS-induced mice colitis		Butyrate and HIF-1α have a mutual regulatory mechanism, maintaining of barrier function	Caleb J. Kelly et al. (46)	2015
	DSS-induced mice colitis		*F. prausnitzii* produces butyrate to maintain Th17/Treg balance and ameliorate colitis by inhibiting HDAC1 and IL-6/STAT3/IL-17 pathway	Lixing Zhou et al. (38)	2018
	DSS-induced mice colitis		NLRP1 aggravates colitis by limiting beneficial butyrate-producing *Clostridiales*, reversed by butyrate supplementation	Hazel Tye et al. (31)	2018
	TNBS-induced colitis mice	Caco-2 cells	Sodium butyrate ameliorated intestinal epithelium barrier dysfunction through activating GPR109A and inhibiting the AKT and NF-κB p65 signalling pathways.	Guangxin Chen et al. (47)	2018
	Clostridium difficile-Induced Colitis		Butyrate attenuates intestinal inflammation and improves intestinal barrier by activation of HIF-1.	José Luís Fachi et al. (48)	2019
		Organoids derived epithelial cells	Butyrate does not protect against inflammation-induced loss of epithelial barrier function and cytokine production.	Maaike Vancamelbeke et al. (49)	2019
	DSS-induced mice colitis		EHLJ7 enhances butyric acid production, cooperates with butyrate to inhibit JAK2/STAT3/SOCS1 pathway, improving UC symptoms	Xiaonan Tang et al. (32)	2020
	DSS-induced mice colitis		HSF2 might induced by sodium butyrate and inflammation and played protective roles in UC by enhancing autophagy of IECs.	Fengrui Zhang et al. (50)	2020
	DSS-induced mice colitis		Butyrate can alleviate DSS-induced colitis by regulating autophagy via HIF-1α.	Chao Zhou et al. (51)	2020
		Ex vivo differentiated epithelial organoids (d-EpOCs)	Intestinal inflammation alters the response of the epithelium to butyrate, particularly under the influence of tumour necrosis factor-alpha (TNFα), making it less responsive to butyrate during active inflammation	Elena Ferrer-Picón et al. (43)	2020
	DSS-induced mice colitis		Butyrate reverses CYP2A activity decrease in colitis-induced mice, highlighting microbiota’s role in regulation	Stefan Satka et al. (33)	2022
	DSS-induced mice colitis		Butyrate from *Clostridium butyricum* stimulated EGFR activation in colon, balancing the inflammatory cytokines, protecting tight junctions, and increasing the number of goblet cells and MUC2 production.	Jingyi Wu et al. (52)	2022
	DSS and Carrageenan-induced mouse model		D-Met and BA supplementation attenuates disease conditions and suppresses inflammation-related gene expressions in DSS-induced colitis mouse model.	Yuka Ikeda et al. (30)	2023
3-Hydroxybutyrate	DSS-induced mice colitis		Poly-D-3-hydroxybutyric acid, a slow-release 3-HB donor, suppresses IBD pathogenesis by upregulating regulatory T cells	Rimina Suzuki et al. (41)	2023
n-butyrate	DSS-induced mice colitis		N-butyrate upregulates intestinal claudin-23 expression through SP1 and AMPK pathways and enhances barrier function.	Wenxi Xu et al. (53)	2023
Acetate		Organoid-derived epithelial monolayer cultures	High acetate protects intestinal barrier, reduces inflammation, and upregulates barrier genes in UC patient-derived organoids.	Sara Deleu et al. (34)	2023

SCFAs, Short-chain fatty acids.

### Inflammation and immune regulation

SCFAs, particularly butyrate, exhibit anti-inflammatory properties. Among SCFAs, butyrate is most extensively studied related to inflammation and immune regulation in IBD. Acting on receptors GPR43 and GPR109a, butyrate effectively suppresses the development of IBD (28, 29), and its supplementation reduces the expression of inflammation-related genes in UC (30). Nonetheless, the activation of the inflammasome sensor Nlrp1 triggers IL-18, which paradoxically inhibits the benefits of butyrate-producing bacteria, exacerbating inflammation in UC (31). EHLJ7, a quaternary coptisine derivative, not only stimulates butyrate production but also collaborates with butyrate to inhibit the JAK2/STAT3/SOCS1 signalling pathway, ultimately alleviating UC symptoms (32). Prophylactic administration of butyrate can reverse the reduced activity of cytochrome P450 2A5 induced by UC, a phenomenon associated with the gut microbiota (33). In addition to butyrate, acetate supplementation decreases levels of pro-inflammatory cytokines such as IL-8 and TNF-α in UC-derived epithelial cells while simultaneously upregulating hypoxia-inducible factor (HIF1α) and mucin 2 (34). Valproic Acid, functioning as a histone deacetylase (HDAC) inhibitor, elevates H3K27ac levels in UC mice, effectively suppressing the production of inflammatory cytokines (35).

Maintaining the balance of T cells has always been a hot spot for the exploration of the pathogenesis of IBD. Dendritic cells exposed to butyrate can induce the differentiation of naïve T cells into Foxp3-expressing Treg cells (36). Moreover, SCFAs can directly enhance Foxp3 expression by activating GPR43 on the surface of T cells (37). A butyrate producer, *Faecalibacterium prausnitzii*, fosters forkhead box protein P3 (Foxp3) expression by inhibiting HDAC1, thereby disrupting the IL-6/STAT3/IL-17 pathway and altering the Treg/Th17 equilibrium (38, 39). The administration of a probiotic combination comprising *Saccharomyces boulardii*, *Lactobacillus rhamnosus*, *Lactobacillus acidophilus*, and *Bifidobacterium breve* to SAMP1/YitFc mice resulted in increased SCFAs levels and gene expression changes involved in memory B cell development and T cell infiltration (40). Employing poly-D-3-hydroxybutyric acid as a controlled-release agent for 3-hydroxybutyrate, sustained butyrate release enhances regulatory T cell presence (41).

While considerable research has delved into the anti-inflammatory effects of butyrate, challenges persist in its application, with some IBD patients exhibiting suboptimal responses to butyrate treatment (42). Investigations have revealed that the reduction of butyrate-producing bacteria in the feces of IBD patients is unrelated to the decrease in butyrate concentration, and the sensitivity of IBD patients to butyrate remains unchanged, but the elevation of TNF-α diminishes the response of intestinal epithelial cells to butyrate (43). This “disabling” effect of butyrate during active UC might be linked to altered gene expression patterns in UC (44) or possibly associated with compromised regulation of the CTLA-4 receptor on T cell surfaces induced by butyrate (45). These studies could potentially offer novel insights for enhancing the application of SCFAs.

### Regulation of gut microbiota

Dysbiosis of the gut ecosystem stands as a significant hallmark in IBD, with beneficial probiotics producing SCFAs being recognized for their favorable impact on IBD. Conversely, the proliferation of certain pathogenic bacteria contributes to intestinal inflammation associated with disease onset and relapse (54). Among these probiotics, butyrate-producing bacteria have garnered attention for their capacity to maintain mucosal barriers, regulate immune functions, and alleviate inflammation (55). A reduction in butyrate-producing bacteria, including *Clostridium coccoides*/*Eubacterium rectale*, *Clostridium leptum*, and *Faecalibacterium prausnitzii* in the intestinal lumen, as well as *Roseburia* spp. in the mucosa of UC patients (56). The abundance of *Lachnospiraceae*is significantly diminished regardless of active or remission phase of UC (57). Apigenin mitigates DSS-induced colitis by modulating *Akkermansia*, *Turicibacter*, *Klebsiella*, *Romboutsia* (58). Conversely, *Paraclostridium bifermentans* exacerbates UC symptoms, potentially linked to SCFAs reduction (59). The expansion of gut microbiota in UC patients can stimulate the occurrence of cancer through the activation of T helper cell types 1 and 17 cytokines (60). Acetate suppresses uvrY-dependent type 1 pilus expression and hinders the adherence of invasive Escherichia coli (61).

Dietary fiber serves as a source of SCFAs, and diets rich in dietary fiber are advantageous for the amelioration of inflammatory conditions (62). A Cohort study discovered that dietary fiber intake can reduce the occurrence of CD exacerbations (63). Conversely, the lack of dietary fiber prompts a shift in the gut microbiota’s nutritional preference to mucin glycans, detrimentally impacting mucosal epithelium (64). Soluble dietary fiber from quinoa bran increases tight junction protein expression, enhances gut microbial diversity, boosts SCFAs production, and alleviates DSS-induced colitis (65). Similarly, highly purified insoluble dietary fiber extracted from soybean bran exhibits similar effects (66). However, A case-control study found that dietary fiber had a limited impact on microbial and SCFAs profiles (67).

### Maintenance of intestinal barrier

SCFAs play a pivotal role in maintaining intestinal barrier integrity. Prior research has indicated that HIF coordinates intestinal protection (46). Butyrate reduces damage to the intestinal barrier inflicted by *Clostridium difficile* (48), regulates intestinal epithelial cell autophagy (51), modulates tight junction (74) through a HIF-dependent mechanism, and thereby alleviates DSS-induced colitis. The stabilization of HIF by butyrate is hypothesized to occur through the inhibition of HIF prolyl hydroxylase (75).

The maintenance of tight junction components is a critical factor in protecting the intestinal barrier. Among these, butyrate takes center stage in SCFAs (47). Reduced tight junction protein 1 (TJP1) expression and elevated claudin-1 expression characterize the intestinal epithelium of IBD patients. *Butyricicoccus* can upregulate claudin-1 expression, and maintain the integrity of intestinal epithelial tight junctions (76). Butyrate might also enhance tight junction protein abundance by activating the AKT/mTOR pathway (77). Heat shock transcription factor 2 can inhibit mTOR and promote butyrate-induced autophagy, thereby protecting intestinal epithelial cells (50). Isobutyric acid upregulates claudin-23 protein through the SP1 and AMPK pathways (53). Butyrate-producing bacteria also activate epidermal growth factor receptors (EGFR), contributing to tight junction protection (52). However, research also pointed out that co-culturing butyrate with TNF-α and IFN-γ can lead to epithelial damage, suggesting potential contrasting effects of butyrate in IBD (49).

Besides, exploration of acetate and propionate has enriched the spectrum of SCFAs effects on IBD. Acetate, exhibiting lower epithelial toxicity, maintained the epithelial barrier and reduced levels of pro-inflammatory factors such as IL-8 and TNF-α (34). Propionate enhanced the expression of tight junction proteins zonula occludens-1 (ZO-1) and occludin, which exerted anti-inflammatory effects by inhibiting macrophages (78). Furthermore, propionate upregulated endothelial cell-selective adhesion molecules, reinforcing intestinal epithelial tight junctions (79).

Supplementing bacteria that produce butyrate for CD patients (80), or administering butyrate derivatives orally (81), can achieve protection of the intestinal barrier. The intricate relationship between gut microbiota and intestinal barrier function is pivotal, where probiotics can mitigate epithelial damage and regulate inflammatory responses. The combination of *Faecalibacterium prausnitzii*, a butyrate-producing bacterium, with chitosan oligosaccharides, can restore tight junction levels and enhance intestinal epithelial barrier function (82). Supplementation with prebiotics, probiotics, IgG, and amino acids collectively improves IBD intestinal barrier function (83). Soluble dietary fiber from quinoa bran (65) and Panax quinquefolius polysaccharides both elevate tight junction protein expression, subsequently augmenting SCFAs production.

### SCFAs as biomarkers and potential therapeutic targets

A notable feature of IBD patients is the reduced levels of SCFAs, evident in feces, urine, plasma, intestinal fluid, and exhaled gas, highlighting the potential of SCFAs as diagnostic and predictive biomarkers for IBD remission and relapse. Total SCFAs in rectal fluid are reduced in recurrent IBD patients (106). Butyrate levels in feces decrease during active CD and UC phases, implying its potential as a marker for active IBD (107). The diminished capacity for butyrate synthesis is more pronounced in CD than in UC (22). Regardless of relapse status, IBD patients have lower isobutyrate levels than healthy individuals (106). Furthermore, isobutyrate and *Verrucomicrobiota* could serve as markers for diagnosing UC remission (108). Additionally, hexanoate might serve as a predictive indicator for CD patient relapse risk (25). Targeted delivery of SCFAs to the colon alleviated inflammation in DSS-induced colitis mice, enhancing SCFAs application for IBD (109).

## Medium-chain fatty acids

MCFAs are usually defined as fatty acids with carbon chain lengths ranging from 7 to 12 carbon atoms. As directly absorbable fatty acids, MCFAs facilitate quick energy supply, promote intestinal mucosal repair, and regulate inflammation (132). The colon plays a pivotal role in the digestion and absorption of MCFAs. Moreover, MCFAs exhibit inherent antibacterial effects, and maintain gut microbiota balance by reducing pathogenic bacteria and promoting beneficial bacteria (132).

The role of MCFAs in patients suffering from UC and CD has been elucidated by various epidemiological investigations. Observations from a case-control study have demonstrated significantly reduced levels of lauric acid (C12:0) in the serum of patients with autoimmune diseases (133). MCFAs such as heptanoate, octanoate and nonanoate, exhibit markedly decreased levels in IBD patients (134). In pediatric patients, there were variations of lauric acid between CD and UC, and higher levels of lauric acid were observed during UC active phases compared to remission (135). In summary, reduced levels of MCFAs are common among IBD patients, and proportions of MCFAs differ between CD and UC.

MCFAs alleviates IBD symptoms by regulating inflammation and intestinal epithelial barrier (**Table 2**). Jun Wang et al. investigated the impact of octanoic acid and nonanoic acid on intestinal mucosal barrier. They found that treatment with these acids enhanced intestinal barrier function and reduced bacterial translocation by reducing activity of histone deacetylases, resulting in increased secretion of porcine β-defensin 1 and porcine β-defensin 2 (69). Octanoic acid can also serve as a molecular conjugate to enhance the activity of other drugs. The novel drug CLX-103, composed of mesalazine, eicosapentaenoic acid, and octanoic acid, shows prolonged intestinal retention and greater efficacy compared to mesalazine alone (68). Decanoic acid can induce increased paracellular permeability, influencing tight junctions in intestinal epithelial cells (70). Lauric acid has been found to have anti-inflammatory effects in other diseases (136), though its role in IBD remains unclear. Black soldier fly larvae (BSFL) oil, rich in lauric acid, is proposed to modulate mTOR signalling and enhance PPAR target gene expression related to fatty acid oxidation. This demonstrates anti-inflammatory activity and amelioration of colitis symptoms in mice (72). However, several studies have demonstrated the pro-inflammatory activity of MCFAs. MCFAs regulate inflammation in human fetal intestinal epithelial cells by promoting IL-8 secretion induced by IL-1β and TNF-α (137). Additionally, lauric acid could induce NOD2 signalling and increase NF-kB activation and IL-8 expression (71).

**Table 2:** Mechanism of MCFAs in inflammatory bowel disease.

MCFAs	Animal model	Cell/Tissue	Mechanism	Author	Year
Caprylic acid (8:0)	DSS-induced mice colitis		Caprylic acid could form conjugates, increase mesalazine activity and enhance its retention in the intestines.	Mahesh Kandula et al. (68)	2016
Caprylic acid (8:0) and nonanoic acid (9:0)		IPEC-J2 porcine jejunal epithelial cells	Caprylic acid and nonanoic acid reduce bacterial translocation, and significantly increase the secretion of porcine β-defensin 1 (pBD-1) and pBD-2.	Jun Wang et al. (69)	2018
Capric acid sodium (10:0)		Surgical specimens of distal ileum from CD patients or colon specimens from colon cancer patients (control)	Capric acid sodium could induce increased paracellular permeability, affecting intestinal epithelial cell tight junctions.	J D Söderholm et al. (70)	2002
Lauric acid (12:0)		HCT116 human colon epithelial cell line	Curcumin can inhibit lauric acid-induced Nod2 signalling, suppressing NF-kB and IL-8 expression.	Shurong Huang et al. (71)	2008
	DSS-induced mice colitis		The use of BSFL oil can regulate mTOR signalling and promote an increase in PPAR target genes for fatty acid oxidation.	Hadas Richter et al. (72)	2023
Amphiphilic cyclobutene (C10) and Cyclobutane cis-C18 fatty acid		Map K-10	Amphiphilic cyclobutene and Cyclobutane cis-C18 fatty acid can inhibit Mycobacterium avium subspecies paratuberculosis (Map) associated with Crohn’s disease.	Denise K Zinniel et al. (73)	2019

MCFAs have been employed for the prediction and treatment of IBD. Serum lauric acid serves as a significant biomarker for autoimmune diseases, while the relationship between MCFAs and IBD cannot be firmly established due to the limited sample size (133). Medium-chain triglycerides (MCTs) hold potential therapeutic value. However, due to the risk of formulations with MCTs lacking essential PUFAs and fat-soluble vitamins, the use as additives is not recommended for healthy children (138). Additionally, a novel amphiphilic C10 and C18 cyclobutene and cyclobutane fatty acid has demonstrated inhibition of *Mycobacterium avium subsp. paratuberculosis* associated with CD (73, 139).

## Long-chain fatty acids

LCFAs generally encompass fatty acids with carbon atoms ranging from 13 to 22 (8) and are categorized into saturated fatty acids (SFAs), monounsaturated fatty acids (MUFAs), and polyunsaturated fatty acids (PUFAs) based on unsaturation degrees. Dietary intake is a common means of obtaining LCFAs, such as ω-3 and ω-6 fatty acids found in fish oil (140). The significant variations among various LCFAs yield distinct implications for IBD development (**Table 3**).

**Table 3:** Mechanism of LCFAs in inflammatory bowel disease.

	LCFA	Animal model	Cell/Tissue	Mechanism	Author	Year
SFAs	Palmitic acid		Human colonic LS174T goblet cells	Palmitic acid induces altered Muc2 secretion due to ER stress in goblet cells, potentially exacerbating IBD.	Quentin Escoula et al. (84)	2019
			Caco-2 cells	Palmitic acid affects intestinal epithelial barrier integrity and permeability in Crohn’s disease.	Manuele Gori et al. (85)	2020
			Caco-2 cells	Palmitic Acid induces NF-κB pathway and downstream cytokines.	Romina Bashllari et al. (86)	2023
MUFAs	Oleic acid		Human Intestinal smooth muscle Cell	Oleic acid does not affect IL-8 production in Crohn’s-derived intestinal smooth muscle cells, potentially having no impact on inflammation.	M A Alzoghaibi et al. (87)	2003
		DSS-induced rat colitis		Diet rich in oleic acid from acorn-fed ham alters gut microbiota, reduces colitis symptoms and inflammation, and enhances antioxidant activity.	J Fernández et al. (88)	2020
		DSS-induced mice colitis		High intake of extra virgin olive oil or flaxseed oil does not prevent DSS-induced colitis in mice and may cause adverse effects.	Roberto de Paula do Nascimento et al. (89)	2020
	Oleic acid (Oleic acid is a major component of Brucea javanica oil)	DSS-induced mice colitis		Oleic acid-rich Brucea javanica oil emulsion exhibits anti-inflammatory effects by inhibiting NF-κB activation in DSS-induced colitis in mice.	Yan-Feng Huang et al. (90)	2017
	9- or 10-nitro-octadecenoic oleic acid	DSS-induced mice colitis		Nitrated oleic acid activates PPARγ, reduces colonic inflammation, and improves symptoms of DSS-induced colitis.	Sara Borniquel et al. (91)	2010
PUFAs	ω-3 fatty acid	TNBS-induced rat colitis		Dietary ω-3 fatty acid decreases PEG2 and AP, therefore, ameliorates inflammation and mucosal damage in experimental ulcerative colitis.	Natalia Nieto et al. (92)	2002
		Transgenic mice biosynthesizing		Increased tissue ω-3 PUFA leads to anti-inflammatory resolvin formation, reducing NF-κB activity, TNF-α, IL-1β, and inducible NO synthase.	Christian A Hudert et al. (93)	2006
		TNBS-induced rat colitis		Dietary n−3 PUFA attenuates inflammation in TNBS-induced rat colitis via PPAR−γ/NFAT pathway.	Jiayin Yao et al. (94)	2017
	ALA	Rat colitis models (DSS, TNBS)	Human Caco-2 cells	ALA-rich sage oil ameliorates colitis by down-regulating pro-inflammatory genes, including IL-6, COX2, TNFα, IL-8, and iNOS.	Ram Reifen et al. (95)	2015
		DSS-induced mice colitis		ALA alleviates DSS-induced colitis in mice by suppressing colon damage and inflammation, by reducing the expression of ionized calcium binding adaptor molecule 1-positive macrophages.	Jeongtae Kim et al. (96)	2020
	Eicosapentaenoic acid	Acetic acid-induced rat colitis		EPA mitigates AA-induced colitis by modulating TGF-β/P-EGFR and NF-κB pathways, balancing oxidant/antioxidant, and enhancing colon barrier integrity.	Raghda N El Mahdy et al. (97)	2023
	Polyunsaturated fatty acids	Mouse models, Xbp1-/-IEC and Gpx4+/-IEC	Human CD epithelial organoids	PUFA excess induces ER stress, activates IRE1α via TLR2, triggers chemokine production, and therefore exacerbates CD.	Julian Schwärzler et al. (98)	2022
	ω-6 polyunsaturated fatty acids	DSS-induced mice colitis		ω-6 PUFA in Western diet triggers GPX4-restricted mucosal inflammation resembling colitis via cytokine response and impaired GPX4 activity.	Lisa Mayr et al. (99)	2020
	ω-6 polyunsaturated fatty acids (linseed oil, extruded linseed)	DSS-induced mice colitis		ω-6 polyunsaturated fatty acids in linseed oil and extruded linseed rebalancing ω-6/ω-3 PUFA ratio, gut dysbiosis, inflammation, and microbiota modulation.	Claire Plissonneau et al. (100)	2022
	DPA	DSS-induced mice colitis		DPA attenuates inflammation in DSS-induced colitis model; modulates pro-inflammatory cytokines (TNF-α, IL-1β, IL-6) and anti-inflammatory cytokine (IL-10); inhibits synthesis of pro-inflammatory eicosanoids (PGE2, LTB4).	Zhenxiao Zheng et al. (101)	2019
		DSS-induced mice colitis		DPA supplementation enriches the diversity of gut microbiota and increases butyrate production.	Ye Dong et al. (102)	2022
	DHA, EPA		LS174T goblet cells	DHA and EPA alleviate palmitic acid-induced ER stress in LS174T goblet cells, protect Muc2 secretion, has potential therapy for lipotoxicity.	Quentin Escoula et al. (84)	2019
	ETYA		Human Intestinal Organoids	ETYA and butyrate regulate ECM genes, suppress collagen content, and reduce tissue stiffness in CD-related strictures.	Ingrid Jurickova et al. (103)	2022
	Icosapent ethyl	Acetic acid-induced rat colitis		Icosapent alleviates acetic acid-induced rat colitis by modulating SIRT1 pathway, reducing inflammation, oxidative stress, and apoptosis.	Ahmed Ahmed Abdelsameea et al. (104)	2023
	17S−epoxy−docosapentaenoic acid	DSS-induced mice colitis		DiHEP-DPA reduces inflammation, decreases pro-inflammatory cytokines, and inhibits NF-κB pathway in UC model.	Lifang Wang et al. (105)	2022

DPA, Docosapentaenoic acid; ALA, Alpha-Linolenic acid; DHA, Docosahexaenoic acid; EPA, Eicosapentaenoic acid; ETYA, Eicosatetraynoic acid.

### Saturated fatty acids and trans fatty acid

SFAs have been identified to exhibit pro-inflammatory effects. Interestingly, trans fatty acids (TFAs), a distinct subset of unsaturated fatty acids, seem to induce similar responses to SFAs (141). Both SFAs and TFAs potentially regulate inflammatory responses by impacting the PPAR-γ and retinoid X receptor (RXR) pathways (142). Despite the fact that the precise roles of SFAs and TFAs in IBD remains enigmatic, extensive studies have shown their contribution to IBD onset and progression. A genetic study focusing on fatty acid profiles found that C18 TFAs, total TFAs, and palmitic acid may positively correlate with the onset of IBD (143). Several epidemiological studies also demonstrated that higher intake of SFAs and TFAs is associated with elevated UC risk (4, 5), whereas a meta-analysis did not observe a significant correlation between SFA intake and IBD (144). Furthermore, a diet rich in TFAs could exacerbate pathological inflammation in the intestinal epithelium of individuals with IBD (94). In addition, a multicenter prospective study found that consuming high intake of myristic acid increased the risk of UC recurrence in remission (145). Another cohort study indicated a significant difference in urinary TFAs among patients with UC recurrence (146).

Palmitic acid is a key component of SFAs, which is associated with pro-inflammation. Plasma metabolomics measurements suggested that palmitic acid was a potential diagnostic marker for IBD (147). Palmitic acid contributes to inflammation by enhancing intestinal epithelial permeability (85), activating NF-κB pathway and cytokines (86), and inducing endoplasmic reticulum stress leading to intestinal epithelial cell lipotoxicity (84).

### Monounsaturated fatty acids

MUFAs, characterized by a single double bond, have been observed to possess anti-inflammatory properties in animal experiments (148, 149). However, investigations on the effects of MUFAs on IBD are limited and contradictory. S. Bühner et al. identified a reduction in MUFAs (18:1, ω-9) levels by analyzing fatty acid profiles of biopsies from CD patients (150), while another study reported increased oleic acid levels in IBD patients (14). Mounting case-control studies have associated increased MUFA intake with elevated UC incidence (4, 151). In the case of palmitoleic acid, a study revealed elevated serum levels in CD patients, correlating with increased surgical intervention risk (152). Conversely, a cross-sectional analysis found lower MUFA intake in CD patients (153), and a meta-analysis incorporating four case-control and five prospective studies did not establish a significant association between MUFAs and UC incidence (144). In conclusion, further epidemiological and clinical research is required to elucidate the role of MUFAs in IBD.

As one of the prominent MUFAs, oleic acid has garnered considerable attention from researchers. Early experiments indicated decreased levels of oleic and palmitoleic acids in the inflamed intestinal mucosa (154). Oleic acid exerts a protective effect on CD patient intestinal epithelial cells without upregulating interleukin-8 (IL-8) levels (87). Furthermore, oleic acid-containing Javanese Bruise Oil Emulsion and ellagic acid-containing thymol polyphenols can inhibit the NF-κB pathway in DSS-induced colitis mice (90, 155). In addition, 9- or 10-nitrooctadec-9-enoic acid activates the PPAR-γ pathway, displaying superior anti-inflammatory efficacy in IBD compared to natural oleic acid (91). Mice consuming oleic acid-rich acorn-fed ham exhibited enhanced anti-inflammatory microbial abundance and cecal SCFAs concentration after DSS induction, suggesting potential preventive effects against UC (88). On the contrary, excessive consumption of oleic acid-rich extra virgin olive oil failed to ameliorate symptoms in DSS-induced colitis mice and even elevated TNF-α levels (89). Therefore, MUFAs potentially serve as an anti-inflammatory fatty acid with preventative and therapeutic roles in IBD. Nonetheless, contradictions across studies necessitate further experimental exploration into the specific mechanisms underlying MUFAs’ actions in IBD.

### Polyunsaturated fatty acids

PUFAs comprise a category of fatty acids characterized by multiple double bonds. Among the various PUFAs, ω-6 fatty acids promote IBD progression (14), whereas ω-3 fatty acids possess anti-inflammatory properties (13). High ω-6/ω-3 fatty acid ratio in Western diets is a pivotal feature of IBD.

ω-6 fatty acids include arachidonic acid (AA) and linoleic acid, which are often associated with promoting UC pathogenesis (14). Accumulating evidence from population-based revealed an increased level of AA in the colonic mucosa of UC patients (154) associated with IBD-related colorectal cancer progression (156, 157). Metabolomic analysis indicated perturbed AA metabolism in UC mucosa (158), and an increased likelihood of ω-6 fatty acid depletion in pediatric IBD patients (159). Observations from a cohort inquiry highlight a positive correlation between dietary AA intake and UC risk (160). A Mendelian analysis proposed ω-6 fatty acids as causative for CD (161).

ω-3 fatty acids exert anti-inflammatory effects and are often considered protective against IBD. Diminished levels of ω-3 fatty acids were observed in both CD and UC patients (162). A Japanese case-control study suggested that PUFA intake serves as a protective factor for UC, while excessive docosahexaenoic acid (DHA), eicosapentaenoic acid (EPA), and docosapentaenoic acid (DPA) intake might increase UC risk (163). Conversely, two Mendelian analyses independently associated ω-3 fatty acids with protection against IBD (164, 165). A nurse cohort study found a connection between ω-3 fatty acid intake and reduced UC risk (5). Meta-analytic investigations revealed protective effects of fish oil and dietary ω-3 fatty acids against UC (13). Conversely, a systematic review highlighted that supplementing PUFAs has a negligible impact on IBD prevention and treatment, with limited long-term inflammation improvement (166). Another scoping review revealed inconclusive effects of ω-3 fatty acids on IBD inflammation and symptom alleviation (167).

PUFAs can impact IBD through influencing intestinal inflammation. Elevated AA levels in UC patient’s colonic mucosa are linked to inflammation and colonic mucosal phospholipid AA composition (154, 168). PUFAs can modulate anti-inflammatory signalling pathways and maintain the integrity of the intestinal barrier by binding to GPR120 (169, 170). The anti-inflammatory effects of ω-3 fatty acids were linked to decreased levels of prostaglandin E2 (PGE2), alkaline phosphatase (AP) (92), and the downregulation of NF-κB, TNF-α, inducible nitric oxide synthase, and IL-1β activity (93), along with upregulation of PPAR-γ (94). Alpha-linolenic acid (ALA) could reduce ionized calcium-binding adaptor molecule 1-positive macrophages (96) and downregulate the expression of pro-inflammatory genes IL-8, cyclooxygenase-2 (COX2), and inducible nitric oxide synthase to reduce intestinal inflammation (95). EPA moderates TGF-β/P-EGFR and NF-κB inflammatory pathways, modulates the redox balance, and mitigates rat UC progression (97). Recent research revealed the antioxidative activity of glutathione peroxidase 4 against PUFA-induced oxidative stress. Excessive dietary PUFAs could activate toll-like receptor 2 (TLR2) through inositol-requiring enzyme 1α (IRE1α)-induced endoplasmic reticulum stress (98), and AA might trigger cytokine production akin to ferroptosis in intestinal epithelial cells, leading to epithelial injury (99).

Microbial dysbiosis and impaired mucosal barrier are pivotal features in IBD pathogenesis, and PUFAs might influence IBD by altering microbial balance and maintaining mucosal barrier. Flaxseed supplementation and ω-6-rich fatty acids have been associated with promoting microbial dysbiosis in CD and altering mucosal barrier functions (100, 171). Supplementation of DPA increases gut microbial diversity and changes microbial composition in DSS-induced colitis mice (102). Furthermore, DHA and EPA alleviate endoplasmic reticulum stress in goblet cells, reducing the synthesis and secretion of Muc2 protective mucin barriers (84). Limited evidence suggested DPA possesses stronger anti-inflammatory and protective effects against IBD than EPA and DHA (101), offering new directions for PUFAs research.

Eicosatetraynoic acid (ETYA) (20:4) in a patient-specific human intestinal organoid (HIO) model developed by Ingrid Jurickova et al. demonstrated reduced extracellular matrix gene expression and alleviated CD ileal stricture (103). Icosapent ethyl can mitigate CD ileitis through the silent information regulator 1 (SIRT1) pathway, decreasing the expression of various pro-inflammatory cytokines and exerting anti-inflammatory, antioxidant, and anti-apoptotic effects (104). Lifang Wang et al. synthesized 7S, 15R-dihydroxy-16S, 17S-epoxy-22-carbon-5-docosenoic acid and demonstrated its anti-inflammatory effects by reducing TNF-α, IL-6, and IL-1β levels (105). Notably, traditional Chinese herbal formulas may modify IBD progression by regulating PUFAs (172–174).

Researches have extensively been exploring the regulation of inflammation and intestinal barrier by PUFAs. Recent studies elucidate PUFAs mechanisms impacting IBD progression via ferroptosis-related pathways and the influence of PUFAs on microbial ecology, thereby expanding perspectives in IBD research.

## Very long-chain fatty acids

The role of VLCFAs, defined as fatty acids with a carbon chain length exceeding 23, is gradually being elucidated in inflammation. VLCFAs can originate from dietary sources and can also be endogenously synthesized from long-chain fatty acids and Elongated by the elongase of very long fatty acid (ELOVL) family (175). Saturated VLCFAs can mediate necroptosis (176) and activate macrophages to generate an inflammatory response (177). However, research on VLCFAs has primarily focused on X-linked adrenoleukodystrophy, a disorder characterized by VLCFAs accumulation. There is a gap in understanding of VLCFAs’ role in IBD.

Research on VLCFAs in IBD is limited. Only some gene sequencing revealed differences in the ELOVL gene between IBD patients and healthy individuals. Genetic sequencing of two CD patients in Japan revealed variations in ELOVL6 (178), and gene analysis of immune cells from UC patients demonstrated significant upregulation of ELOVL5 (179). Furthermore, ELOVL7 gene regions were identified as possible novel loci associated with adalimumab response in CD patients (180). Additionally, octacosanol was demonstrated to alleviate DSS-induced colitis by protecting intestinal barrier and modulating gut microbiota and SCFAs levels (181).

## Lipid derive mediators

Lipid mediator (or oxylipins) is a group of bioactive molecules that derive from fatty acid involving at least one step of dioxygen-dependent oxidation (10). PUFAs serve as major precursors for lipid mediators. Inflammatory responses and resolution are perceived as a controlled, ongoing process involving sequential release of diverse lipid mediators. Prostaglandins (PGs) and leukotrienes (LTs) derived from AA are pro-inflammatory mediators at inflammation onset, while lipoxin A4 (LXA4) and LXB4 from AA and ω-3 fatty acids derived lipid mediators are recognized as pro-resolving mediators during resolution. Furthermore, the interaction of a novel lipid mediator endocannabinoid system with exogenous cannabinoids is considered to be a potential therapeutic avenue for IBD (182). Lipid mediators are increasingly recognized for their significance in the pathophysiology and treatment of IBD (**Table 4**).

**Table 4:** Mechanism of lipid mediators in inflammatory bowel disease.

Lipid Mediators	Animal Model	Cell	Mechanism	Author	Year
PGE2	TNBS-induced mice colitis		PGE2 shifts IL-12/IL-23 balance, promoting IL-23, and exacerbating IBD.	Amir F Sheibanie et al. (110)	2007
Lipocalin-type PGD synthase	DSS-induced mice colitis		Increased L-PGDS expression was found in active UC patients, and L-PGDS exacerbated mice colitis.	Ryota Hokari et al. (111)	2011
PGD2 receptors	DSS-induced mice colitis		DP and CRTH2 receptors have opposing roles in DSS-induced mice colitis inflammation regulation.	Eva M Sturm et al. (112)	2014
PGD2	TNBS-induced mice colitis		CRTH2 activation in eosinophils contributes to CD inflammation, while CRTH2 antagonist reduces inflammation.	Balázs Radnai et al. (113)	2016
	DSS-induced mice colitis		Niacin increases PDG2 releases and reduce inflammation.	Juanjuan Li et al. (114)	2017
LTB4	TNBS-induced mice colitis		BLT1 (LTB4 receptor) in dendritic cells promotes Th1/Th17 differentiation, exacerbating colitis via cytokine modulation.	Jinfeng Zhou et al. (115)	2018
LXA4	Cyclooxygenase 2 (Cox2) total knockout and myeloid knockout (MKO) mice		Administration of an LXA4 analog rescued disease in Cox2-MKO mice.	David Meriwether et al. (116)	2019
RvE1	DSS-induced mice colitis		RvE1 induces intestinal alkaline phosphatase (ALPI) expression, detoxifies LPS, and promotes inflammatory resolution.	Eric L Campbell et al. (117)	2010
	TNBS-induced mice colitis		RvE1 derived from omega-3 eicosapentaenoic acid (EPA) reduces leukocyte infiltration and counter-regulates pro-inflammatory gene expression to protect mice from colitis.	Makoto Arita et al. (118)	2005
	DSS-induced mice colitis		RvE1 derived from eicosapentaenoic acid inhibits pro-inflammatory cytokines, regulates macrophage responses via ChemR23, and ameliorates colonic inflammation in colitis model.	Tsukasa Ishida et al. (119)	2010
RvD1	DSS-induced mice colitis		RVD1 remodelling gut microbiota, restoring intestinal barrier integrity, reducing inflammation, and improving gut-liver axis communication.	Cui Zeng et al. (120)	2022
	DSS-induced mice colitis		RvD1 reduces autophagy-induced EMT in intestinal epithelial cells and ameliorates intestinal fibrosis by disrupting epithelial-fibroblast crosstalk.	Cui Zeng et al. (121)	2022
PD1, RvD5	DSS-induced mice colitis		PD1 and RvD5 protect against colitis and inflammation by reducing leukocyte adhesion and emigration.	Thomas Gobbetti et al. (122)	2017
MaR1	Mouse models (DSS-induced colitis, TNBS-induced colitis)		MaR1 attenuates colonic inflammation by inhibiting NF-κB pathway and inflammatory mediators, reducing neutrophil migration, and enhancing macrophage M2 phenotype.	Rodrigo Marcon et al. (123)	2013
	DSS-induced colitis rat		MaR1 reduces inflammation by reducing neutrophil and macrophage infiltration, activating Nrf2 signalling, and inactivating TLR4/NF-κB signalling. Besides, MaR1 improves TJ protein expression and decreases MPO and ROS activity, therefore ameliorating DSS-induced colitis.	Shujin Qiu et al. (124)	2020
5-HETE		Differentiated Caco-2 cells	Leukotriene D4 and 5-Hydroxyeicosatetraenoic acid alter paracellular permeability and epithelial barrier function by activation of the phospholipase C/Ca (2+)/protein kinase C pathway and cAMP-independent protein kinase A activation.	M J Rodríguez-Lagunas et al. (125)	2013
12-HETE	DSS-induced mice colitis		Systemic Alox15 (leukocyte-type 12-LOX) deficiency suppresses the formation of 12-hydroxyeicosatetraenoic acid (12-HETE), reduced expression of pro-inflammatory gene products, and sustained expression of ZO-1.	Saskia Kroschwald et al. (126)	2018
15- HEPE	DSS- and TNBS-induced colitis in fat1 transgenic mice with endogenously increased n-3 PUFA tissue status		Alox15 deficiency suppresses the formation of n-3 PUFA-derived 15-HEPE. Intraperitoneal injections of 15S-HEPE protect mice from DSS- and TNBS-induced colitis.	Nadine Rohwer et al. (127)	2021
15- HETE		Human Caco-2 cells	EGCs from CD patients have reduced 15-HETE production, leading to impaired control of IEB permeability via inhibition of adenosine monophosphate-activated protein kinase and increased expression of ZO-1.	Camille Pochard et al. (128)	2016
Endocannabinoid anandamide	Chronic ileitis model (TNFΔARE/+ mice)		CB2R activation on Tregs enhances suppressive function and IL-10 secretion, attenuating murine ileitis by improving histological scoring and decreasing inflammatory cytokine expression.	Kristina L Leinwand et al. (129)	2017
endocannabinoid	DSS-induced mice colitis		Diet high in linoleic acid dysregulates the intestinal endocannabinoid system and increases susceptibility to colitis in mice.	Nathan Calzadilla et al. (130)	2023
Endocannabinoidome (eCBome) lipid mediators: LEA, OEA, etc.	TNBS-induced mice colitis		Depletion of gut microbiota and subsequent differential development of the gut immune system in germ-free mice leads to altered eCBome lipid mediator levels, contributing to reduced susceptibility of mice to DNBS-induced colitis.	Tommaso Venneri et al. (131)	2023

PGE2, Prostaglandin E2; PGD2, Prostaglandin D2; LTB4, Leukotriene B4; LXA4, Lipoxin A4; RvE1, Resolvin E1; RvD1, Resolvin D1; PD1, Protectin D1; MaR1, Maresin 1; HETE, Hydroxyeicosatetraenoic acid.

### Arachidonic acid derived lipid mediators

Lipid mediators derived from AA primarily include PGs, LTs, and LXA4. A case-control study has identified a series of pro-inflammatory lipid mediators, such as PGE2, PGD2, and thromboxane (TXB2) in the intestinal mucosa of UC patients, correlated with the severity of inflammation (183). Interestingly, PGD2 also exhibited a protective effect, and its upregulation was associated with prolonged remission in UC patients (183). Elevated LXA4 expression promotes mucosal homeostasis and can be found only in the intestinal mucosa during remission (184). EPA, an AA metabolite generated through P450 metabolism, has been observed at elevated levels in UC tissues in comparison to the surrounding tissues, suggesting a potential protective role against UC (185). Evidence from observational study points to higher levels of AA-derived oxylipins in CD patients (186).

PGs serve as key regulators of vascular responses, modulating inflammatory cell infiltration (187). PGE2 and PGD2 play a crucial role in inflammation modulation, originating from the common precursor PGH2 through enzymatic catalysis. In IL-10 knockout mice, PGD2, PGE2, and other lipid mediators are upregulated. This may result from local inflammatory cell induced breakdown of PUFA to maintain inflammatory homeostasis (188). PGE2 exerts pro-inflammatory effects in IBD, possibly through the IL-23/IL-17 axis regulation (110). PGD2 may have both pro-inflammatory and anti-inflammatory effects under different circumstances. For instance, the increased presence of lipocalin-type prostaglandin D synthase (L-PGDS) in UC may exacerbate inflammation (111). Conversely, PGD2 also has protective effects in IBD. Nicotinic acid regulates suppressed macrophage pro-inflammatory gene expression through PGD2 release (114). The effects of PGD2 depend on its receptors; activation of CRTH2 may mediate inflammation, while stimulation of another PGD2 receptor DP is inversely correlated with neutrophil activity to mitigate inflammation (112, 113).

LTs promote leukocyte activation and cytokine production. In trinitrobenzene sulfonic acid (TNBS)-induced colitis mice, LTB4 inhibition accelerates colitis healing. Furthermore, LTB4 synthesis in colonic epithelial cells is enhanced in TNBS-induced colitis mice, which modulates Th1 and Th17 cell differentiation (115). LXA4 and LXB4, anti-inflammatory pro-resolving lipid mediators derived from AA, inhibit vascular inflammation (189). Knocking out COX2 reduces colonic LXA4 levels, exacerbating inflammation (116).

AA derived lipid mediators can also serve as biomarkers and potential therapeutic targets. Due to its significant role in inflammation, the major urinary metabolite of PGE is considered to be a predictive biomarker of mucosal healing and relapse (190). Furthermore, in a lipidomics study of UC patients, PGE1 and PGD2 are critical factors influencing mucosal healing (191). Inhibition of LTB4 might hold potential therapeutic implications for UC (192).

### ω-3 fatty acids derived lipid mediators

ALA can be metabolized into DHA and EPA, which are subsequently transformed by LOX and CYP enzymes into various ω-3 fatty acid-derived lipid mediators (193). ω-3 fatty acids derived lipid mediators include resolvins, protectins, and maresins, are considered potential tools for controlling IBD due to their remarkable pro-resolving capabilities, which are also named specialized pro-resolving mediators (SPMs) (194). In an observational study, RvE1 was found to be positively correlated with disease activity in UC patients (195). Resolvins and protectins from ω-3 fatty acids have a significant up-regulation in IBD colon biopsies (122). A case-control study has revealed lower levels of ω-3 fatty acid-derived lipid mediators in CD patients (186).

Resolvins are interactive products generated during the inflammation resolution phase, contributing to alleviating inflammatory stress (10). Protectin, a lipid mediator produced by DHA, exhibits the ability to inhibit neutrophil migration and promote neutrophil apoptosis (196, 197). Maresins, macrophage mediators derived from DHA, demonstrate pronounced anti-inflammatory effects (198). Resolvin E1 (RvE1) derived from EPA induces the expression and activity of alkaline phosphatase in intestinal epithelial cells to detoxify lipopolysaccharide (LPS) (117). RvE1 suppresses pro-inflammatory cytokine TNF-α production, reducing leukocyte-mediated tissue damage and gene expression (119), ultimately improving histological scores in colitis mice and preventing colitis onset (118, 119). BLT1 can serve as a receptor for RvE1, accelerating the intestinal epithelial barrier repair (199). RvD1, sourced from DHA, confers protection against IBD by strengthening tight junctions, maintaining intestinal homeostasis, and attenuating inflammation (120). RvD1 also reduces mesenchymal transition of intestinal epithelium and prevents IBD-associated fibrosis (121). Both RvD1 and RvD5 diminish leukocyte adhesion, migration, and endothelial adhesion activated by TNF-α, which exhibit protective effects against colitis (122). Maresin 1 diminishes IL-1β, TNF-α, IL-6, and IFN-γ levels and suppresses NF-κB pathway (123). Besides, maresin 1 regulates NRF2 and TLR4/NF-kB pathways to control colonic inflammation, concurrently improves tight junction protein expression, and maintains mucosal barrier (124). Targeted transport of maresin 2 via nanoparticle packaging promotes mucosal repair (200). Besides, DHA-derived epoxides can restore the ability of the endothelium to resolve intestinal inflammation through major facilitator superfamily domain containing 2A (MFSD2A), and overexpression of MFSD2A reduces colitis in mice (201).

Certain lipid mediators derived from ω-3 fatty acids have the potential to serve as biomarkers reflecting the progression of IBD. Compared to CD, RvE1 levels are significantly elevated in active UC, while during CD remission, protectin DX concentrations are higher than UC (202). However, another study suggests that RvE1 may not be a biomarker for UC (195). Enteral nutrition can stimulate the release of RvD1-RvD5 through innate lymphoid cell activation to alleviate CD symptoms (203).

### Other lipid mediators

Hydroxyeicosatetraenoic acid (HETE) and cannabinoids are important lipid mediators influencing IBD. Accumulating case-control research suggests elevated levels of 5-HETE, 11-HETE, 12-HETE, 15-HETE, and endocannabinoids in inflamed mucosa of IBD (186, 203, 204). Pro-inflammatory AA derivative 15S-HETE elevated in CD and 12S-HETE higher during CD remission (204). Functional variants of cannabinoid receptor 2 increase the risk of pediatric CD (205). Distinct differences exist in the endocannabinoid systems between individuals with IBD and colorectal cancer (206).

HETEs derived from various PUFAs could regulate inflammation. HETE is upregulated in IL-10 knockout mice, for local inflammatory cell induce PUFA breakdown (188). 5-HETE enhances intestinal epithelial cell permeability through the cAMP-independent protein kinase A pathway and phospholipase C/Ca (2+)/protein kinase C pathway (125). Exogenous supplementation of 12-HETE reduces the expression of tight junction protein ZO-1, impairing the intestinal barrier function in UC (126). Conversely, ω-3 supplementation exerts a protective effect on UC by increasing 15-HETE (127, 207). Mechanistic study on 15-HETE reveals its role in reducing intestinal epithelial permeability by inhibiting AMPK and increasing the activity of ZO-1 (128).

Endocannabinoid system has recently been identified to offer protective effects in IBD. Under chronic IBD conditions, the endocannabinoid system undergoes downregulation, while the activation of cannabinoid receptor 2 mitigates inflammation by enhancing regulatory T cell (Treg) function and IL-10 secretion (129). DSS-induced colitis mice exhibit an increase in colonic mucosal endocannabinoids (130). Disruption of the endocannabinoid system in mice by a high ω-6 fatty acid diet heightens susceptibility to UC (208). Recent findings suggest that inflammation induced in germ-free mice by DNBS is attenuated, attributing to the role of the endocannabinoid system in the endocannabinoidome (eCBome) (131).

These lipid mediators also serve as biomarkers and potential therapeutic targets. The 20- HETE-d6 could serve as a diagnostic biomarker for IBD (147). Patients with IBD might self-administer cannabis to alleviate abdominal pain symptoms (209). Phycocyanin has been found to activate cannabinoid receptors and promote UC healing (210).

## Therapeutic approaches based on fatty acids

A myriad of therapeutic strategies is available for IBD, encompassing surgical interventions, dietary interventions, corticosteroid hormone therapy, biologics, fecal microbiota transplantation, probiotic therapy, and enteral nutrition. In this context, we will specifically highlight three treatment approaches closely associated with fatty acids and lipid mediators, including dietary interventions, SCFAs and lipid mediator supplementation.

Biological agents, 5-aminosalicylic acid (5-ASA), glucocorticoids, and immunosuppressive drugs are widely used for induction and maintenance treatment in IBD patients. The therapeutic mechanisms of these drugs are partly related to fatty acids. Butyrate salts might synergistically enhance the therapeutic effects of anti-TNF treatments (211). An increase in SCFA-producing bacterial levels is one of the mechanisms by which Infliximab therapy exerts its therapeutic effect (212), and serves as a biomarker for patient response to Infliximab therapy (213). Levels of butyrate and substrates are correlated with the remission outcomes of Infliximab treatment (214). Similarly, butyric acid and isobutyric acid can serve as biomarkers for patient responses to Vedolizumab (108). Furthermore, glucocorticoids, such as prednisolone, can directly reduce luminal lipid mediators like PGE2 and LTB4, improving IBD symptoms (215, 216). The effects of 5-ASA on UC intestinal mucosa SCFA have been elaborated on in other reviews (217). A recent clinical study found that UC patients treated with a combination of 5-ASA and FEEDColon involving Butyrate achieved better subjective symptom improvement than those treated with 5-ASA alone (218). Additionally, animal studies have found that 1000mg/kg EPA in alleviating colitis-related oxidative stress, serum LDH, colonic GLP-1 expression, and NF-κB capacity is comparable to Sulfasalazine (97). These studies illuminate the role of fatty acids in IBD drug therapy.

### Dietary intervention: particularly the therapeutic role of fatty acids in IBD

Fatty acids play a pivotal role in the pathological and physiological processes of IBD by regulating intestinal mucosal barrier, modulating pro-inflammatory/anti-inflammatory balance, influencing immune function, and regulating gut microbiota homeostasis. Therefore, a rational intake of fatty acids is crucial for both IBD prevention and treatment.

Increasing the intake of ω-3 fatty acids is generally deemed advantageous for IBD. Consumption of anti-inflammatory diets rich in ω-3 fatty acids has been linked to alterations in the gut microbiota of UC patients during remission, resulting in a reduction of subclinical inflammation (219). Incorporating a Japanese diet, which is high in fish oil and has high ω-3 fatty acids, is also beneficial for clinical remission in UC patients (220). ω-3 fatty acids exhibit anti-inflammatory effects in postoperative patients without influencing the risk of postoperative complications (221). The use of EPA has shown promise in lowering calprotectin levels and decreasing UC relapse (222), as well as improving gut microbiota composition and inflammation (223). Supplementing with cis-palmitoleic acid can downregulate the expression of hepatocyte nuclear factor (HNF4α) and HNF4γ, potentially mitigating inflammation in UC patients (224). Moreover, the combination of amino-salicylic acid and ω-3 fatty acids effectively maintains remission in pediatric CD (225).

However, ω-3 fatty acids exhibit contradictory effects. Combined supplementation of ω-3 fatty acids and vitamin D does not significantly improve CD inflammation and clinical remission (226). Multicenter randomized controlled trials have demonstrated the ineffectiveness of using free ω-3 fatty acids for preventing CD relapse (227). Hence, the European Society of Parenteral and Enteral Nutrition suggests a dietary approach characterized by lower ω-6 and higher ω-3 content to prevent IBD, while advising against ω-3 supplementation for maintaining IBD remission (228).

The Mediterranean diet, rich in fish oil and PUFAs and low in SFAs, was considered a relatively healthful dietary pattern (229). It has been recommended as a part of auxiliary treatment for patients with IBD (230). Embracing a healthful lifestyle, such as the Mediterranean diet, has demonstrated potential in ameliorating IBD disease activity and inflammation status (229). Mediterranean diet can elevate the levels of SCFA-producing bacteria, maintaining clinical remission in patients with quiescent UC (230), and reducing the mortality associated with IBD (231). The Mediterranean diet might exhibit superior therapeutic efficacy for mild-to-moderate CD compared to specific carbohydrate-based diets (232).

The composition of fatty acids can also play a guiding role in the proportion of enteral nutrition (EN). Early EN stands as a primary therapeutic choice for CD patients (233), offering a relatively low-cost option. EN formulations contain fewer SFAs than the National Diet and Nutrition Survey (234). Prior research has highlighted the varied impacts of EN with different lipid compositions on CD patients, with higher proportions of oleic acid showing a stronger relief impact compared to higher proportions of stearic acid (235). The ratio of ω-6 to ω-3 fatty acids exhibits a significant positive correlation with CD remission, while total MCFAs and LCFAs show an inverse correlation with remission (236). This insight contributes to a better understanding of optimal EN fatty acid ratios.

### SCFAs supplements

The anti-inflammatory potential of SCFAs has been well established in animal experiments, with clinical trials showcasing their promise in treating IBD. Butyrate enema therapy was previously deemed as an efficacious treatment for distal colitis (237). However, its effectiveness has not been substantiated in large-scale RCTs, and the inconvenience associated with the long-term administration of enemas has led to its gradual phasing out (238). Oral microencapsulated sodium butyrate has been found to lower calprotectin levels, sustain remission, enhance quality of life and promote SCFA-producing bacteria in IBD patients (239). Nevertheless, issues of potential poor tolerance to butyrate salts have been raised (240). The use of an herbal mixture containing chamomile flowers and charcoal has demonstrated an ability to elevate butyrate concentrations in UC patients, resulting in reduced relapses (241). Butyrate salts derived from dietary fiber fermentation have shown promise in raising butyrate levels and improving symptoms like abdominal pain and reflux (242). However, Sunil Thomas et al. suggest caution in supplementing with dietary fiber, as high-fiber diets might amplify immunotherapy-associated toxicity in UC (243). Moreover, the efficacy of sodium butyrate in pediatric IBD appears limited (244). Further extensive research is warranted to further develop therapeutic protocols centered around butyrate interventions.

Probiotic supplementation contributes to rectifying gut microbiota balance and alleviates IBD symptoms through elevated SCFAs. Probiotic supplements also reduced inflammation (244). Intake of fermented vegetable beverages containing *Pediococcus pentosaceus* led to improved loose stool symptoms associated with higher levels of acetic, propionic, and butyric acids (245). A composite formulation of calcium butyrate, *Bifidobacterium bifidum*, *Bifidobacterium lactis*, and fructooligosaccharides improved the quality of life and alleviated abdominal pain and diarrhea (218). Prebiotics can promote the growth and reproduction of beneficial microbacteria. Oral fructooligosaccharide prebiotics rich in inulin augmented butyrate production, lowering UC severity (246).

### Lipid mediator supplements

The endocannabinoid system is a significant lipid mediator, the homeostasis of which can be modulated by exogenous cannabinoids. In UC patients, supplementation with cannabinoids led to significant downregulation of AA, palmitoylethanolamine, and anandamide levels, reshaping the tone of endocannabinoid system and ameliorating UC symptoms (247). Cannabinoid supplementation exhibited improvement in active CD and reduced corticosteroid dependency (248). The exploration of lipid mediator-derived therapies remains underdeveloped, pointing toward a potential avenue for future research.

## Conclusion

In summary, this review encompasses the mechanisms of fatty acid impact on IBD, the current epidemiological landscape, and therapeutic strategies. Fatty acids, as essential dietary components, have been identified to exert anti-inflammatory, immune-regulatory, gut-microbiota-modulating, and barrier-maintaining effects within the pathological processes of IBD. However, the mechanisms of specific fatty acids like MCFAs and VLCFAs in IBD necessitate further elucidation, potentially directing future research endeavors. We have conducted an overview of epidemiological research investigating the impact of fatty acids on IBD. It reveals inconsistencies among certain epidemiological studies and disparities between findings from animal experiments and epidemiological investigations. Furthermore, the availability of robust evidence from large-scale cohorts and randomized controlled trials is limited, and translating results from animal models to human populations presents challenges.

Fatty acids can serve as adjunctive therapeutic tools in managing IBD. We compile existing dietary recommendations and supplementary strategies utilizing fatty acids for therapeutic intents. However, pure fatty acid supplementation often falls short of meeting diverse patient needs. As the effects of each fatty acid and lipid mediator are unveiled, demands for refined dietary strategies or supplement regimens for IBD treatment or relapse prevention intensify. More precise fatty acid ratios and composite formulations may pave the way for future investigations. Additionally, as a disease influenced by genetics and environment, IBD manifests varied sensitivity to different fatty acids or lipid mediators among different genotypes. Leveraging personalized fatty acid supplements based on individual genetic phenotypes through artificial intelligence and extensive data systems could illuminate the path toward tailored interventions.

## Author contributions

DY: Conceptualization, Investigation, Methodology, Writing – original draft, Writing – review & editing. SY: Conceptualization, Data curation, Methodology, Validation, Writing – original draft. YH: Validation, Writing – review & editing. SW: Writing – original draft, Writing – review & editing. YX: Resources, Writing – review & editing. XX: Validation, Writing – review & editing. MD: Resources, Supervision, Writing – review & editing. WL: Data curation, Resources, Writing – review & editing. XC: Conceptualization, Project administration, Supervision, Writing – original draft, Writing – review & editing. XW: Conceptualization, Funding acquisition, Project administration, Supervision, Writing – review & editing.
